# Mortality among Italians and immigrants with COVID-19 hospitalised in Milan, Italy: data from the Luigi Sacco Hospital registry

**DOI:** 10.1186/s12879-022-07051-9

**Published:** 2022-01-19

**Authors:** Andrea Giacomelli, Anna Lisa Ridolfo, Cecilia Bonazzetti, Letizia Oreni, Federico Conti, Laura Pezzati, Matteo Siano, Cinzia Bassoli, Giacomo Casalini, Marco Schiuma, Alice Covizzi, Matteo Passerini, Marco Piscaglia, Fabio Borgonovo, Claudia Galbiati, Riccardo Colombo, Emanuele Catena, Giuliano Rizzardini, Laura Milazzo, Massimo Galli, Antonio Brucato, Spinello Antinori

**Affiliations:** 1 III Infectious Diseases Unit, L. Sacco Hospital, ASST Fatebenefratelli-Sacco, Via G.B. Grassi 74, 20157 Milan, Italy; 2 Department of Biomedical and Clinical Sciences DIBIC, Luigi Sacco, Università Di Milano, Milan, Italy; 3 Infectious Diseases Unit, ASST Fatebenefratelli-Sacco, Luigi Sacco University Hospital, Milan, Italy; 4 Division of Internal Medicine, ASST Fatebenefratelli Sacco, Fatebenefratelli Hospital, Milan, Italy; 5 Department of Anesthesiology and Intensive Care, ASST Fatebenefratelli-Sacco, Luigi Sacco Hospital, Milan, Italy; 6 School of Medicine, University of the Witwatersrand, Johannesburg, South Africa

**Keywords:** SARS-CoV-2, Immigrants, Outcomes, Italy, South America, Europe, Asia, Africa, Country of origin

## Abstract

**Background:**

To compare differences in the probability of COVID-19-related death between native Italians and immigrants hospitalised with COVID-19.

**Methods:**

This retrospective study of prospectively collected data was conducted at the ASST Fatebenefratelli-Sacco Hospital in Milan, Italy, between 21 February and 31 November 2020. Uni- and multivariable Cox proportional hazard models were used to assess the impact of the patients' origin on the probability of COVID-19-related death.

**Results:**

The study population consisted of 1,179 COVID-19 patients: 921 Italians (78.1%) and 258 immigrants (21.9%) who came from Latin America (99, 38%), Asia (72, 28%), Africa (50, 19%) and central/eastern Europe (37, 14%). The Italians were significantly older than the immigrants (median age 70 years, interquartile range (IQR) 58–79 *vs* 51 years, IQR 41–60; p < 0.001), and more frequently had one or more co-morbidities (79.1% *vs* 53.9%; p < 0.001). Mortality was significantly greater among the Italians than the immigrants as a whole (26.6% *vs* 12.8%; p < 0.001), and significantly greater among the immigrants from Latin America than among those from Asia, Africa or central/eastern Europe (21% *vs* 8%, 6% and 8%; p = 0.016). Univariable analysis showed that the risk of COVID-19-related death was lower among the immigrants (hazard ratio [HR] 0.43, 95% confidence interval [CI] 0.30–0.63; p < 0.0001], but the risk of Latin American immigrants did not significantly differ from that of the Italians (HR 0.74, 95% CI 0.47–1.15; p = 0.183). However, after adjusting for potential confounders, multivariable analysis showed that there was no difference in the risk of death between the immigrants and the Italians (adjusted HR [aHR] 1.04, 95% CI 0.70–1.55; p = 0.831), but being of Latin American origin was independently associated with an increased risk of death (aHR 1.95, 95% CI 1.17–3.23; p = 0.010).

**Conclusions:**

Mortality was lower among the immigrants hospitalised with COVID-19 than among their Italian counterparts, but this difference disappeared after adjusting for confounders. However, the increased risk of death among immigrants of Latin American origin suggests that COVID-19 information and prevention initiatives need to be strengthened in this sub-population.

## Background

The Coronavirus Disease 2019 (COVID-19) pandemic caused by the newly identified severe acute respiratory syndrome coronavirus 2 (SARS-CoV-2) started to hit Europe in late February 2020, when there was an abrupt surge in the number of severely and critically ill respiratory patients in northern Italy, particularly Lombardy [1]. From then to week 44 of 2021, 76,799,553 cases and 1,433,500 deaths were reported in the EU/EEA, many of which occurred in Italy [2].

The probability that people infected with SARS-CoV-2 will die is one of the most concerning aspects of the COVID-19 pandemic. Early observational studies of hospitalised COVID-19 patients found that an older age, a higher co-morbidity burden, obesity, and disease severity upon hospital admission all markedly influenced the risk of death [3]. Furthermore, data from ongoing studies of the general population spread of the virus [4] suggest that a higher risk of exposure is associated with socio-economic vulnerabilities, such as limited educational and employment opportunities and/or belonging to an ethnic minority, and that people in poorer general health are more likely to develop severe and fatal illness [5–8]. It has also been observed that immigrants are at greater risk of exposure and infection than native populations, probably because they often work in high-risk occupations, live in overcrowded accommodation, and face barriers to healthcare and prevention initiatives [9, 10]. Some studies have found that immigrants with COVID-19 are also at increased risk of hospitalisation [9, 10], but it is unclear whether this means that they are also at increased risk of COVID-19-related death [11, 12].

The aim of this study was to assess differences in the probability of COVID-19-related death between native Italians and immigrants with COVID-19 admitted to two major hospitals in Milan, Italy.

## Methods

### Study design

This was a retrospective observational study of prospectively collected data relating to a cohort of hospitalised COVID-19 patients.

### Setting

The study was conducted at the Department of Infectious Diseases and the intensive care unit of Luigi Sacco Hospital in collaboration with the Department of Internal Medicine of Fatebenefratelli Hospital. Luigi Sacco Hospital, which is located on the outskirts of Milan, is one of the city’s major infectious diseases centres, and has been at the forefront of the hospitalisation of COVID-19 patients since the first days of the pandemic in Italy [13–16]. The Department of Internal Medicine of Fatebenefratelli Hospital, which is located in the inner city, was rapidly converted to a COVID-19 centre when the pandemic struck.

As laid down in Italian healthcare regulations, urgent and essential healthcare is provided free of charge to Italians and immigrants regardless of their legal status.

### Participants

The study enrolled all of the adult patients with a diagnosis of COVID-19 confirmed by reverse-transcriptase polymerase chain reaction on a nasopharyngeal swab who were admitted to our hospitals between 21 February and 31 November 2020; observation of the cohort was censored on 28 February 2021.

### Data source and management

The characteristics of the data management have been fully described elsewhere [13–16]. In brief, the data were extracted from the patients' clinical charts on a daily basis, and were stored in an ad hoc database. The collected data were the patients’ date and place of birth, and biological sex; the time between symptom onset and hospital admission; co-morbidities (including diabetes, lung diseases, heart diseases, renal diseases, immune system diseases, liver diseases, and obesity defined as a body mass index of ≥ 30) [17]; the burden of co-morbidities (0, 1, 2, and 3 +); whether there was a need for supportive oxygen therapy upon hospital admission; disease severity upon hospital admission (defined as mild, moderate, severe or critical in accordance with the World Health Organisation (WHO) guidelines for the management of COVID-19) [13, 18]; and hospitalisation outcome (date and cause of death, discharge, or transfer to another facility). The vital status of the patients discharged or transferred before the censoring date was ascertained by means of telephone calls.

### Outcomes and variables

The main outcome of interest was COVID-19-related mortality, and the principal variable of interest was place of birth. The patients were classified as natives (if they were born in Italy) or immigrants (sub-divided into four regions of origin: central/eastern Europe, Africa, Latin America, and Asia).

The baseline variables known to be clinically relevant to the outcome of interest [3, 13, 14] and included in the analysis as potential confounders were age, biological sex, the number of days between symptom onset and hospital admission, co-morbidities (including obesity), and disease severity upon hospital admission.

### Statistical analysis

The descriptive statistics of the categorical variables are given as proportions, and those of the continuous variables as median values and interquartile range (IQR). The baseline demographic and clinico-epidemiological characteristics of the Italians and immigrants were compared using the χ^2^ or Fisher's exact test for the categorical variables, and Wilcoxon’s rank-sum test for the continuous variables; the characteristics of the immigrants from different regions of origin were also compared in the same way.

The Kaplan–Meier method was used to plot the survival curves of the Italians and the immigrants as a whole or stratified on the basis of their region of origin (central/eastern Europe, Africa, Latin America, and Asia). Survival curves adjusted for the potential confounders of age, biological sex, time from symptom onset, obesity, and disease severity upon hospital admission were generated using Cox’s model.

The association between the patients’ origins and the risk of COVID-19-related death was investigated using uni- and multivariable Cox proportional hazard ratios (HRs) and their 95% confidence intervals (CIs). All of the variables were entered in the multivariable model by adjusting the effect of immigrant status and region of origin for all of the other co-variates as potential confounders. The discriminative ability of the model was assessed using the integrated area under the receiver-operating characteristic curve (AUC-ROC), which averages all of the available AUC statistics over time. The presence of multi-collinearity among the explanatory variables was verified using the generalised variance inflation factor (GVIF) tool.

All of the statistical analyses were made using SAS software, version 9.4, and differences with a *P* value of < 0.05 were considered statistically significant.

The study was approved by our Ethics Committee (Comitato Etico Interaziendale Area 1, Milan, Italy). All of the patients signed a written informed consent form except for those undergoing mechanical ventilation upon admission for whom it was allowed to be waived.

## Results

**Fig. 1:**
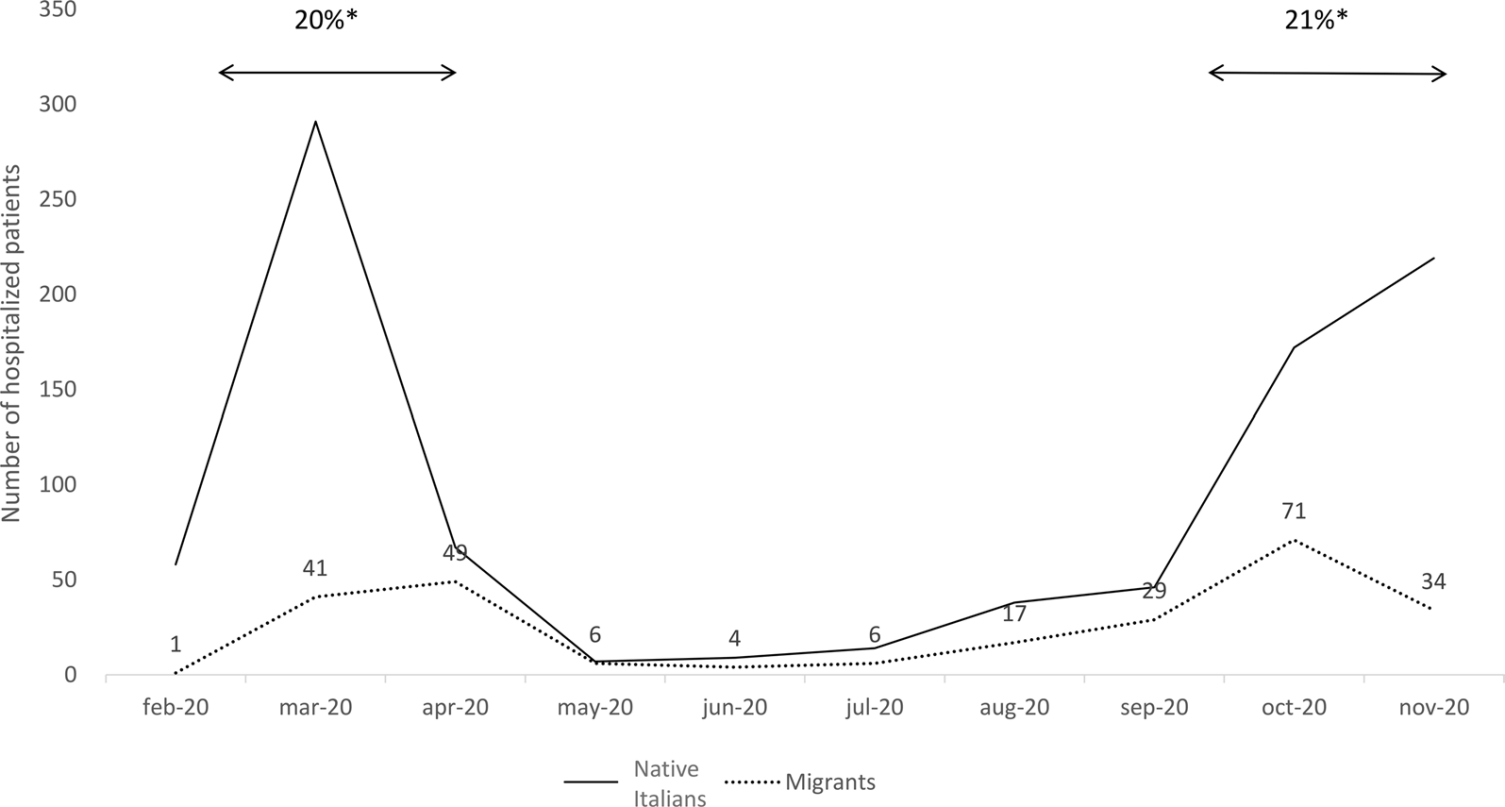
Number of hospital admissions to the Department of Infectious Diseases and intensive care unit of Luigi Sacco Hospital by patient origin. * The percentage of immigrants admitted during the first and second waves

Between 21 February and 31 November 2020, our clinical centres admitted 1,179 COVID-19 patients: 921 Italians (78.1%) and 258 immigrants (21.9%). Figure 1 shows monthly enrolment during the study period: there was no difference in the proportion of immigrants enrolled during the first wave of the pandemic (21 February-30 April) and the second (1 October-31 November).

Table 1 shows the patients’ baseline demographic and clinical characteristics. The Italians were significantly older than the immigrants (median age 70 years, IQR 58–79 *vs* 51 years, IQR 41–60; p < 0.001), and more frequently had one or more co-morbidities (79.1% *vs* 53.9%; p < 0.001), particularly cardiovascular (60.6% *vs* 29.5%) and oncological diseases (14.5% *vs* 6.9%) (p < 0.001 for both). The median time between the onset of COVID-19 symptoms and hospital admission was shorter among the immigrants (6 days, IQR 3–9 *vs* 7 days, IQR 3–19; p = 0.026), but there was no between-group difference in disease severity upon hospital admission.

**Table 1:** Characteristics of the study population by origin

	**Overall** **n = 1179**	**Non-Italians** **n = 258**	**Italians** **n = 921**	**p-value**
Median age [IQR]	65 [53, 76]	51 [41, 60]	70 [58, 79]	< 0.001
Age ≥ 65 years, n (%)	585 (49.6)	40 (15.5)	545 (59.2)	< 0.001
Biological sex				
Female, n (%)	400 (33.9)	86 (33.3)	314 (34.1)	0.882
Male, n (%)	779 (66.1)	172 (66.7)	607 (65.9)	
Co-morbidities				
Obesity^a^, n (%)	231 (19.6)	60 (23.3)	171 (18.6)	0.110
Diabetes, n (%)	166 (14.1)	32 (12.4)	134 (14.5)	0.419
Lung diseases, n (%)	199 (16.9)	29 (11.2)	170 (18.5)	0.006
Heart diseases, n (%)	634 (53.8)	76 (29.5)	558 (60.6)	< 0.001
Renal diseases, n (%)	116 (9.8)	22 (8.5)	94 (10.2)	0.479
Oncological diseases, n (%)	151 (12.8)	17 (6.6)	134 (14.5)	< 0.001
Immune system diseases, n (%)	90 (7.6)	19 (7.4)	71 (7.7)	0.999
Liver diseases, n (%)	38 (3.2)	13 (5.0)	25 (2.7)	0.072
Number of co-morbidities				
0	311 (26.4)	119 (46.1)	192 (20.8)	< 0.001
1	377 (32.0)	68 (26.4)	309 (33.6)	
2	304 (25.8)	41 (15.9)	263 (28.6)	
3 +	187 (15.9)	30 (11.6)	157 (17.0)	
Median number of days from symptom onset [IQR]	7 [3, 10]	6.00 [3, 9]	7 [3, 10]	0.026
Disease severity upon hospital admission^b^				
Mild	113 (9.6)	31 (12.0)	82 (8.9)	0.242
Moderate	487 (41.3)	113 (43.8)	374 (40.6)	
Severe	279 (23.7)	54 (20.9)	225 (24.4)	
Critical	300 (25.4)	60 (23.3)	240 (26.1)	

*IQR* interquartile range

^a^Obesity defined as a body mass index of ≥ 30 [17]

^b^WHO disease severity classification [18]: mild = mild clinical symptoms, no imaging signs of pneumonia; moderate = fever, cough, dyspnea or other symptoms, imaging signs of pneumonia; severe = any of respiratory distress with a respiratory rate (RR) of ≥ 30 breaths per minute; resting oxygen saturation in air ≤ 93%; PaO2 / FiO2 ≤ 300 mmHg); critical = any of respiratory failure requiring mechanical ventilation; shock; any other organ failure needing intensive care

Ninety-nine of the immigrants (38.4%) came from Latin America (mainly from Peru, Ecuador, and El Salvador); 72 (27.9%) from Asia (mainly from The Philippines, Bangladesh, and China); 50 (19.4%) from Africa (mainly from Egypt and Morocco); and 37 (14.3%) from central/eastern Europe (mainly Ukraine, Albania, and Romania). Table 2 shows the differences in the demographic and clinical characteristics of the immigrants by region of origin. The patients from central/eastern Europe included more women (51%) than the other groups (p = 0.015). The patients from Latin America were characterised by a non-statistically significant higher prevalence of obesity and a longer time interval between symptom onset and hospital admission than the other non-Italians (p = 0.015), and less frequent diagnoses of mild disease upon admission (p = 0.011).

**Table 2:** Characteristics of non-Italian patients with COVID-19 by origin

	**Latin Americans** **n = 99**	**Asians** **n = 72**	**Africans** **n = 50**	**Central/eastern Europeans** **n = 37**	**p-value**
Median age [IQR]	50 [42, 58]	51 [36, 59]	53 [41, 63]	54 [46, 63]	0.128
Biological sex					
Female, n (%)	37 (37)	17 (24)	13 (26)	19 (51)	0.015
Male, n (%)	62 (63)	55 (76)	37 (74)	18 (49)	
Co-morbidities					
Obesity^a^, n (%)	31 (31)	10 (14)	10 (20)	9 (24)	0.059
Diabetes, n (%)	11 (11)	9 (12)	8 (16)	4 (11)	0.841
Lung diseases, n (%)	13 (13)	6 (8)	8 (16)	2 (5)	0.339
Heart diseases, n (%)	20 (20)	24 (33)	19 (38)	13 (35)	0.074
Renal diseases, n (%)	8 (8)	9 (12)	3 (6)	2 (5)	0.502
Oncological diseases,n (%)	8 (8)	5 (7)	2 (4)	2 (5)	0.801
Immune system diseases, n (%)	10 (10)	1 (1)	4 (8)	4 (11)	0.137
Liver diseases, n (%)	6 (6)	3 (4)	2 (4)	2 (5)	0.929
Number of co-morbidities					
0	46 (46)	34 (47)	22 (44)	17 (46)	0.992
1	26 (26)	19 (26)	14 (28)	9 (24)	
2	16 (16)	10 (14)	7 (14)	8 (22)	
3 +	11 (11)	9 (12)	7 (14)	3 (8)	
Median number of days from symptom onset [IQR]	7 [4, 10]	6 [2, 8]	4 [2, 8]	5 [3, 7]	0.015
Disease severity upon hospital admission^b^					
Mild	5 (5)	10 (14)	9 (18)	7 (19)	0.011
Moderate	39 (39)	33 (46)	27 (54)	14 (38)	
Severe	30 (30)	8 (11)	7 (14)	9 (24)	
Critical	25 (25)	21 (29)	7 (14)	7 (19)	

*IQR* Inter Quartile Range

^a^Obesity defined as a body mass index of 30 [17]

^b^WHO disease severity classification [18]: mild = mild clinical symptoms, no imaging signs of pneumonia; moderate = fever, cough, dyspnoea or other symptoms, imaging signs of pneumonia; severe = any of respiratory distress with a respiratory rate (RR) of ≥ 30 breaths per minute; resting oxygen saturation in air ≤ 93%; PaO_2_ / FiO_2_ ≤ 300 mmHg); critical = any of respiratory failure requiring mechanical ventilation; shock; any other organ failure needing intensive care

### COVID-19-related mortality

Two hundred and seventy-eight of the 1,179 patients (23.5%) died in hospital within a median of 12 days after admission (IQR 6–20 days). The mortality rate was higher among the Italians (245/921, 26.6%) than among the immigrants as a whole (33/258, 12.8%) (p < 0.001). However, the mortality rate was higher among the immigrants from Latin America (21%) than among those from Asia (8%), central-eastern Europe (8%) or Africa (6%) (p = 0.016), and this difference remained after adjusting for potential confounders (p = 0.028).

**Fig. 2:**
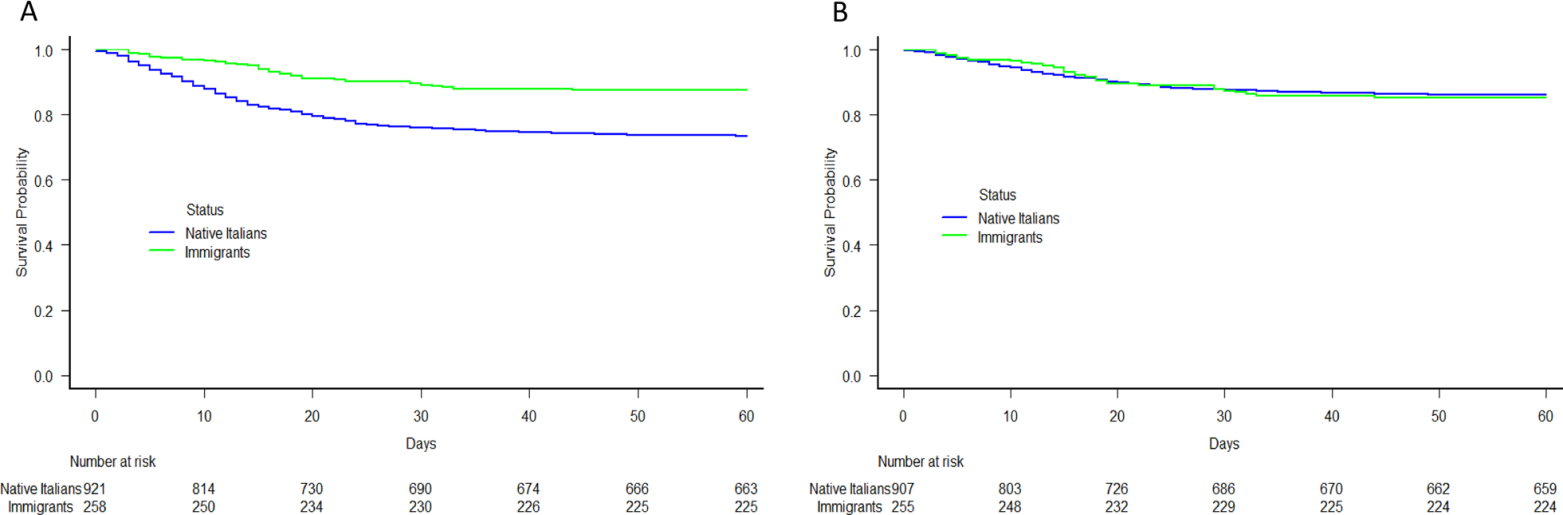
**A** Kaplan–Meier survival curves by patient origin (native Italians *vs* immigrants). **B** Survival curves generated by means of a Cox model (adjusted for age, biological sex, time from symptom onset, obesity, and disease severity upon hospital admission)

Figure 2A and B show the Kaplan–Meier and adjusted survival curves of the Italians and immigrants. The overall probability of COVID-19-related death within 30 days of hospital admission was higher among the Italians: 24%, 95 CI: 21–27% *vs* 11%, 95 CI: 8–15%.; however, after adjusting for age, biological sex, time from symptom onset, obesity, and disease severity upon hospital admission, there was no between-group difference in 30-day mortality.

**Fig. 3:**
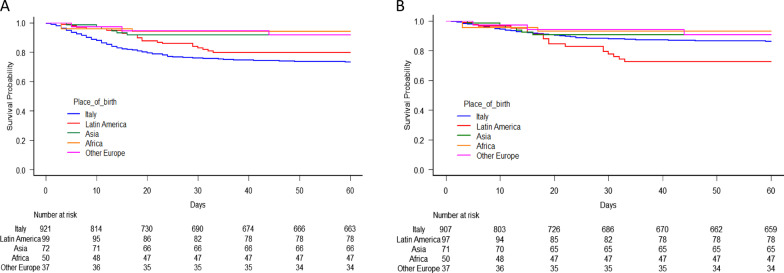
**A** Kaplan–Meier survival curves by patient origin (native Italians *vs* immigrants from Latin America *vs* immigrants from Asia *vs* immigrants from Africa *vs* immigrants from central/eastern Europe). **B** Survival curves generated by means of a Cox model (adjusted for age, biological sex, time from symptom onset, obesity, and disease severity upon hospital admission)

Figures 3A and B show the Kaplan–Meier and adjusted survival curves of the patients by region of origin. Latin Americans had the second highest probability of dying within 30 days of hospital admission (17%, 95 CI: 10–25%) but, after adjusting for age, biological sex, time from symptom onset, obesity, and disease severity upon hospital admission, it was the highest.

### Uni- and multivariable Cox proportional hazard models assessing the effect of immigrant status and region of origin on the risk of COVID-19-related death

Table 3 shows the results of the uni- and multivariable analyses of the factors associated with COVID-19-related death.

**Table 3:** Cox regression analysis of the demographic and clinical factors associated with mortality due to SARS-CoV-2 infection

	**HR**	**95% CI**	**p-value**	**AHR**^**a**^	**95% CI**	**p-value**
Age (per 1 year more)	1.05	1.04–1.06	< 0.0001	1.07	1.06–1.08	< 0.0001
Males *vs* females	1.17	0.90–1.50	0.234	1.41	1.08–1.85	0.011
Time from symptoms onset (per 1 day more)	1.00	0.98–1.02	0.823	0.99	0.97–1.01	0.464
Non Italians *vs* Italians	0.43	0.30–0.63	< 0.0001	1.04	0.70–1.55	0.831
Obesity^b^ YES *vs* NO	1.38	1.05–1.81	0.021	1.67	1.25–2.22	< 0.001
Moderate *vs* Mild COVID-19^c^	2.29	1.11–4.75	0.026	2.05	0.98–4.27	0.056
Severe *vs* Mild COVID-19^c^	3.98	1.92–8.29	< 0.001	3.84	1.82–8.09	< 0.001
Critically *vs* Mild COVID-19^c^	7.56	3.70–15.46	< 0.0001	8.59	4.12–17.88	< 0.0001

*HR* hazard ratio, *CI* confidence interval

^a^Adjusted for age, biological sex, time from symptoms onset, obesity and disease severity at hospital admission

^b^Obesity defined as body mass index of ≥ 30 points [17]

^c^WHO disease severity classification [18]: mild = mild clinical symptoms, no imaging signs of pneumonia; moderate = fever, cough, dyspnea or other symptoms, imaging signs of pneumonia; severe = any of respiratory distress with a respiratory rate (RR) of ≥ 30 breaths per minute; resting oxygen saturation in air ≤ 93%; PaO_2_ / FiO_2_ ≤ 300 mmHg); critical = any of respiratory failure requiring mechanical ventilation; shock; any other organ failure needing intensive care

The crude risk of COVID-19-related death among the immigrants was lower than among the Italians (HR 0.43, 95% CI 0.30–0.63; p < 0.0001). Moreover, the risk of COVID-19-related death among the immigrants from Africa (HR 0.20, 95% CI 0.06–0.63; p = 0.006), Asia (HR 0.28, 95% CI 0.12–0.63; p = 0.002), and central/eastern Europe (HR 0.27, 95% CI 0.09–0.84; p = 0.024) was lower than that of the Italians, whose risk was not significantly different from that of the Latin Americans (HR 0.74, 95% CI 0.47–1.15; p = 0.183) (Table 4).

**Table 4:** Cox regression analysis of the demographic and clinical factors associated with mortality due to SARS-CoV-2 infection

	**HR**	**95% CI**	**p-value**	**AHR**^**a**^	**95% CI**	**p-value**
Age (per 1 year more)	1.05	1.04–1.06	< 0.0001	1.07	1.06–1.08	< 0.0001
Males *vs* females	1.17	0.90–1.50	0.234	1.46	1.12–1.92	0.006
Time from symptom onset (per 1 day more)	0.99	0.98–1.02	0.823	0.99	0.97–1.01	0.408
African *vs* Italian origin	0.20	0.06–0.63	0.006	0.51	0.16–1.61	0.248
Asian *vs* Italian origin	0.28	0.12–0.63	0.002	0.68	0.30–1.54	0.354
Central/eastern Europe *vs* Italian origin	0.27	0.09–0.84	0.024	0.66	0.21–2.07	0.475
Latin American *vs* Italian origin	0.74	0.47–1.15	0.183	1.95	1.17–3.23	0.010
Obesity^b^ yes *vs* no	1.38	1.05–1.81	0.021	1.64	1.23–2.20	0.001
Moderate *vs* mild COVID-19^**c**^	2.29	1.11–4.75	0.026	2.03	0.97–4.24	0.059
Severe *vs* nild COVID-19^**c**^	3.98	1.92–8.29	0.0001	3.76	1.78–7.93	0.001
Critical *vs* mild COVID-19^**c**^	7.56	3.70–15.46	< 0.0001	8.52	4.09–17.76	< 0.0001

*HR* hazard ratio, *CI* confidence interval

^a^Adjusted for age, biological sex, time from symptoms onset, obesity and disease severity at hospital admission

^b^Obesity defined as body mass index of ≥ 30 points [17]

^c^WHO disease severity classification [18]: mild = mild clinical symptoms, no imaging signs of pneumonia; moderate = fever, cough, dyspnea or other symptoms, imaging signs of pneumonia; severe = any of respiratory distress with a respiratory rate (RR) of ≥ 30 breaths per minute; resting oxygen saturation in air ≤ 93%; PaO_2_ / FiO_2_ ≤ 300 mmHg); critical = any of respiratory failure requiring mechanical ventilation; shock; any other organ failure needing intensive care

However, when the Cox model was adjusted for possible confounders, there was no significant difference in the risk of death between the immigrants and the Italians (adjusted HR [aHR] 1.04, 95% CI 0.70–1.55; p = 0.831). Moreover, being of Latin American origin was independently associated with an increased risk of COVID-19-related death (aHR *vs* Italians 1.95, 95% CI 1.17–3.23; p = 0.010). The multivariable analyses also confirmed that age (aHR 1.07 per 1 year more, 95% CI 1.06–1.08; p < 0.0001), male biological sex (aHR: 1.46, 95% CI 1.12–1.92; p = 0.006), obesity (aHR: 1.64, 95% CI 1.23–2.20; p = 0.001), and disease severity upon hospital admission (severe disease (HR 3.76, 95% CI 1.78–7.93; p = 0.001; critical disease: aHR 8.52, 95% CI 4.09–17.76; p < 0.0001) were all independently associated with a higher risk of COVID-19-related death.

The integrated AUC-ROC values of the final model were 0.828 (Italians *vs* immigrants as a whole) and 0.824 (Italians *vs* immigrants stratified on the basis of their region of origin), and the GVIF values did not indicate the presence of multi-collinearity among the independent variables included in the final model.

## Discussion

Just over 20% of our hospitalised COVID-19 patients were immigrants and, excluding the early days of the study period (when the patients were mainly Italians from the first epidemic hotspots in Lombardy) [1, 13], the percentage was similar during the first and second waves of the epidemic in early spring and autumn 2020. This is slightly higher than the percentage of immigrants living in the metropolitan area of Milan (14.1%) or in the inner-city (18.2%) [19], and included a much higher proportion of Latin Americans (38.4%) than the proportion of Latin Americans in the city’s immigrant population as a whole (16.5%) [20]. The fact that the proportion of immigrants in our population of hospitalized COVID-19 patients was higher than the proportion of immigrants living in the metropolitan area of Milan suggests a greater spread of SARS-CoV-2 infection among the city’s immigrant communities. This hypothesis is supported by a recent cross-sectional study of Pagani et al., who assessed the prevalence of anti-SARS-CoV-2 nucleocapsid antibodies among 2,044 Italian and non-Italian inhabitants of a social-housing neighbourhood in Milan. The overall prevalence was 12.4%, but there was a more than two-fold difference between the non-Italians and Italians (23.3% *vs* 9.1%) that was possibly due to various socio-economic and cultural factors [21]. Although it cannot be excluded that immigrants living in Milan are more exposed to SARS-CoV-2 than Italians, it can be speculated that the immigrants’ risk of hospitalisation and death due to COVID-19 is partially mitigated by their younger age [22]: the immigrants in our cohort were significantly younger and less frequently affected by age-related co-morbidities than the Italians, which reflects the demographic differences between the two groups in northern Italy [22]. An older age and age-related co-morbidities are known to be strongly associated with increased COVID-19-related morbidity and mortality [3, 13, 14], and so it is not surprising that in-hospital mortality was greater among the Italians than among the immigrants as a whole or that this difference disappeared after adjusting for the potential confounders of age, biological sex, co-morbidities, and disease severity upon hospital admission. However, it was worryingly unexpected to find that, after adjusting for the same potential confounders, Latin Americans were at higher risk of dying than the Italians and the immigrants from other regions.

The findings of two previous studies of Spanish and immigrant COVID-19 patients hospitalised in Spain (including a majority of immigrants from Latin America) are different: one did not find any significant difference in mortality between patients of European and non-European origin [12], and the other found that mortality was actually lower among the immigrants [11]. The foreign-born patients in these studies had similar demographic characteristics to those of our immigrant patients, but the lack of additional information concerning the clinical drivers of COVID-19 outcomes (such as disease severity or the prevalence of obesity) makes it difficult to make a more detailed comparison. It is also worth remembering that, although Italy and Spain are the main European destinations of Latin American migrants [23], it is likely that those settling in Spain are more integrated, not least because of their common language and cultural proximity.

It is possible that the Latin Americans in our study experienced severe/critical disease more frequently than immigrants from other regions because they knew less about or underestimated the early signs of COVID-19 and were therefore less likely to seek medical advice promptly, or because they may have been afraid of losing wages or their often precarious and unregistered jobs. Such factors were found to be common in a qualitative study of Latin Americans with COVID-19 who were hospitalised in San Francisco [24], and they may explain the excess burden of morbidity and mortality among Latin Americans (particularly more recent immigrants) in the USA [25, 26].

Government policies limiting the access of uninsured or undocumented immigrants to healthcare services can also affect the care-seeking behaviour of immigrants and, although Italy guarantees free emergency healthcare regardless of legal status, undocumented immigrants are highly vulnerable and may be unaware of their rights [27].

Another striking characteristic of our Latin American patients is the high (30%) prevalence of obesity, which substantially increases the risk of COVID-related death [13, 14]. The rates of obesity have markedly increased in Latin America over the last 10–15 years, and it is now considered a public health problem in most Latin American countries [28–31]. In addition, there is evidence that the change in dietary habits associated with immigration and integration increases susceptibility to obesity [29].

The greater frequency of severe COVID-19 and disease-related mortality among the Latin American immigrants hospitalised in two clinical centres in Milan is alarming, and there is a real need to clarify whether it is due to a higher incidence of SARS-CoV-2 infection in the Latin American community, or to cultural, behavioural and socio-economic reasons preventing them from promptly seeking healthcare, or to other factors that have not yet been identified.

### Study limitations

This study has a number of limitations. First of all, its design means that our findings may not apply to different settings and, although the study centres were located in different parts of Milan, it is possible that the study population did not reflect the demographics of the entire metropolitan area. Secondly, the relatively small number of immigrants in our cohort may have limited our characterisation of the differences between groups of immigrants of different origin. Thirdly, given the observational and exploratory nature of this study, no multiplicity adjustment of type I errors was used for the analysis of the main outcome. Finally, we were unable to collect data regarding the patients' educational level or health literacy, their occupations, the length of time they had been in Italy, or their legal status, all of which would have allowed a more precise analysis of the possible association between socio-economic factors and COVID-19 outcomes.

## Conclusions

We found that the mortality rate among immigrants hospitalised with COVID-19 was lower than that of their Italian counterparts. This difference may be due to the fact that the immigrants were significantly younger and less affected by age-related comorbidities than the Italians. However, we did not expect to find that the outcome of Latin American patients with COVID-19 was not only worse than that of their Italian counterparts, but also worse than that of the patients who had immigrated from other regions. This strongly suggests that there is a need for more tailored preventive initiatives, including more information about the manifestations of COVID-19, the testing process, when and where to seek care, and the available vaccinations. It also indicates the importance of continuing research into the factors that play a role in the disparities of COVID-19-related morbidity and mortality in specific populations affected by socio-economic inequalities.

## Data Availability

The datasets used during the current study are available from the corresponding author upon reasonable request.

## Abbreviations

COVID-19: Coronavirus Disease 2019
SARS-CoV-2: Severe acute respiratory syndrome coronavirus 2
WHO: World Health Organisation
IQR: Interquartile range
HR: Cox proportional hazard ratio
CI: Confidence interval
AUC-ROC: Area under the receiver-operating characteristic curve
aHR: Adjusted hazard ratio
GVIF: Generalised variance inflation factor
